# Comparative Study of Different Acupoints for Treating Acute Myocardial Ischemia in Mice

**DOI:** 10.1007/s12265-022-10346-6

**Published:** 2023-01-23

**Authors:** Hao Hong, Xin Cao, Xiang-Min Meng, Qiu-Yu Pang, Li-Juan Zhu, Shu-Guang Yu, Bing-Mei Zhu

**Affiliations:** 1 Regenerative Medicine Research Center, West China Hospital, Sichuan University, Keyuan Road 4, Gaopeng Street, Chengdu, Sichuan 610041 People’s Republic of China; 2 Dushu Lake Hospital Affiliated to Soochow University, Chongwen Road 9, Suzhou, 215000 Jiangsu China; 3 Acupuncture and Tuina School/Third Teaching Hospital, Chengdu University of Traditional Chinese Medicine, Shierqiao Road 37, Chengdu, 610075 Sichuan China

**Keywords:** Acupuncture, Myocardial infarction, Inflammation, Fibrosis, Acupoint specificity

## Abstract

Acupuncture point specificity has been recognized as a key scientific issue in traditional Chinese medicine (TCM), but there is limited clinical trial or animal study to verify the characteristics of PC6, BL15, and ST36 in the protection from myocardial injury. We aimed to compare the effects among these three acupoints on the acute myocardial infarction mice model and to explore possible mechanisms for the first time. We found that PC6 is the most appropriate acupoint to deliver efficacy and safety to treat acute MI in mice. BL15 stimulation improved the systolic function, but increased the risk of arrhythmia. ST36 only slightly attenuated systolic function and had no effect on arrhythmia during MI. RNA profiles of skin tissue in local acupoints demonstrated that the most altered DEGs and related pathways may partly support its best effects of PC6 treatment on MI injury, and support the observed phenomenon of the acupoint specificity.

**Graphical abstract:**

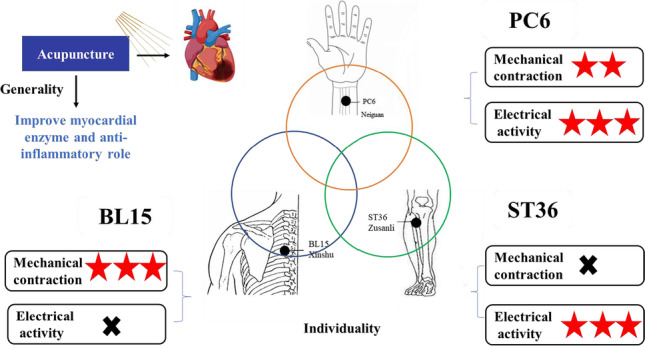

**Supplementary Information:**

The online version contains supplementary material available at 10.1007/s12265-022-10346-6.

## Introduction

Ischemic heart disease (IHD) is a considerable health burden worldwide with high mortality and morbidity, and is the primary cause of disability adjusted life years (DALYs) [1]. Inflammation is implicated in the development of cardiac repair after MI. Although inflammatory signaling is suspected to amplify ischemic injury, inflammatory pathways play an important role in the enlargement and fibrosis remodeling of the infarcted heart [2].

Acupuncture, as a complementary and alternative therapy for IHD-related diseases, has been used to relieve angina and arrhythmias in China for thousands of years in clinical practices and animal studies [3–5]; in addition, acupoints in disease-affected meridian showed a greater reduction of angina attacks than non-affected meridians [3]. However, few studies focus on the difference of acupoint on disease-affected meridian. Acupuncture can alleviate the inflammatory response effectively [6]. A recent study showed that acupuncture stimulation can induce vagal-adrenal anti-inflammatory pathways with layers of skin and point specificity [7]. Anti-inflammatory effects of electroacupuncture can mimic the selective agonists and provide therapeutic advantages to reduce inflammation in infectious diseases [8]. Acupuncture can also relieve cardiac inflammation and fibrosis to improve heart function in mice with diabetic cardiomyopathy [9]. Increasing clinical evidence has confirmed that acupuncture treatment, as the most popular alternative therapy, is effective for various immunological diseases, including infections, allergic disorders, and autoimmune syndromes [10].

In traditional Chinese medicine theory, meridian and acupoint specificity has been debated for some time within the acupuncture field [11–13]. Acupuncture at Neiguan (PC6), Xinshu (BL15), and Zusanli (ST36) have been used together as a complementary treatment to treat myocardial ischemia and heart failure in clinic for thousands of years [14], and PC6 is the major acupoint used to treat ischemic heart disease (IHD) since ancient times according to acupuncture classical theory [15]. Numerous studies have observed that the individual acupoint of these three acupoints can protect from myocardial ischemic or ischemia/reperfusion (I/R) injuries [16–18]. A previous study in rats showed that PC6, but not nonacupoint (position located on the tail near rump) displayed a cardioprotective effect in I/R rats [19]. However, there is few clinical trial or animal study that clarifies the difference of the efficacy of these three acupoints respectively. We aimed to compare the effects among PC6 (pericardial meridian acupoint-directly heart divergent), BL15 (“Beishu” acupoint for heart organ- indirectly heart divergent meridian), and ST36 (nonheart-meridian acupoint) on an acute myocardial ischemia mice model, and to illustrate possible mechanisms for the first time. We also expect to provide more reliable laboratory evidence for the clinical acupoints selection in the treatment of heart diseases by acupuncture.

## Material and Methods

### Experimental Animals

Eight-week-old 20 ± 2 g C57BL/6 J male mice (*n* = 40) were supplied by Ensville Biological Technology Co. LTD (Chengdu, Sichuan, China). Mice were housed at a constant temperature (23 ± 1 °C) under a 12/12-h light–dark cycle, with free access to food and water. All procedures were approved by the Ethics Committee for Animal Care and Use of our university (No. 2020267A), and all procedures were conducted in accordance with the guidelines of the National Institutes of Health Animal Care and Use Committee.

### Animal Groups

Mice were randomly allocated to the Sham-operated group (Sham, *n* = 9), the myocardial infarction group (MI group, *n* = 7), Neiguan group (MI + PC6 group, *n* = 8), Xinshu group (MI + BL15 group, *n* = 8), and Zusanli group (MI + ST36 group, *n* = 8). The surgical procedures were followed as previously described [4]. In brief, mice were anesthetized by 3% isoflurane with 99.5% O_2_ and maintained under anesthesia by 1–1.5% isoflurane. The left anterior descending (LAD) coronary artery was permanently ligated using a 7/0 monofilament suture (Shanghai Pudong Jinhuan Medical Instrument Co. Ltd) to induce myocardial ischemia. Ligation was confirmed by the pale appearance of the apex and anterior wall of the left ventricle. After the surgery, mice were placed on a warm cushion until they were awake. Mice in the Sham group were subjected to the same procedure except for the LAD ligation. The lead II electrocardiogram was monitored before and after the operation (arrhythmia incidence and animal survival rates are shown in Supplementary Table 1).

### Acupuncture Intervention

Acupuncture was initiated at PC6, BL15, or ST36 acupoints bilaterally 48 h after the MI operation, and applied once a day with the mice awake, for 7 days in total. Neiguan (PC6) was located at a point 1.5 cm proximal to the palm crease just above the median nerve; Xinshu (BL15) was located at a point 1.5 cm from the spinous process of the fifth thoracic vertebra in the back; Zusanli (ST36) was located at the anterior tibia muscle, approximately 3 cm below the knee joint. The experimental protocol and acupoints location are shown in Supplementary Fig. 1. The acupuncture needles were folded into an “L” shape by hemostatic forceps and inserted into the PC6, BL15, or ST36 acupoint, separated by approximately 5 mm, and fixed with adhesive tape. We also pressed the needles every 5 min to ensure that they would not fall off and to strengthen the stimulation of the acupuncture intervention. The needles were removed after 15 min.

### Electrocardiogram Recording

Electrocardiograms were performed after 7 days of treatment. All mice were anesthetized in the chamber with 3% isoflurane, then carefully positioned on the electrocardiogram (ECG) recording platform and attached with a mask under 1.5% isoflurane. A surface lead II electrocardiogram was obtained. To minimize stress, we completed the electrodes setup and system adjustment within 5 min, and the first 5 min of each mouse assessment was not included in our ultimate analysis. The subsequent 5-min recording ECG were analyzed by Labchart 8.2.3 (AD Instruments, Adelaide, Australia).

### Echocardiography Analysis

After finishing the seven sessions of acupuncture treatment, all mice underwent transthoracic echocardiography under 1% isoflurane anesthesia to characterize the effects of PC6, BL15, or ST36 on cardiac structure and function using a 12-MHz transducer (i13L, Vivi7 Dimension, Boston, GE), equipped with MX550D detector (25–55 MHz) of wide-band frequency-fusion phase-array transducer. The heart was visualized at B mode from a long-axis view. Left ventricle ejection fraction (EF) and fractional shortening (FS) were calculated from the measurements of wall thickness and chamber diameters. Left ventricle posterior wall thickness at diastole (LVPWd) and left ventricle anterior wall thickness at diastole (LVAWd) were measured at M-mode.

### Biochemical Analyses

Blood samples were obtained and centrifuged at 3500 rpm for 10 min at 4 °C and stored at − 80 °C after separating the serum. The serum levels of CK (JM-11532M1), CK-MB (JM-11532M1), LDH (JM-11330M1), cTnT (JM-11710M1), TNF-α (JM-02415M1), INF-γ (JM-02999M1), IL6 (JM-02446M1), IL-10 (JM-02459M1), and renin (JM-02627M1) were measured using commercially available kits (Jiangsu Jingmei Biological Technology Co. Ltd, Nanjing, China) in accordance with the manufacturer’s instructions. The methods and procedures strictly followed the protocols of the test kits.

### Hematoxylin and Eosin Staining

Ischemic heart tissue was dissected and immediately fixed with 4% paraformaldehyde. Hematoxylin and eosin staining were performed on serial Sects. (4 μm thick) of paraffin-embedded heart tissues. Briefly, the sections were dewaxed in xylene, rehydrated in descending grades of ethanol, and washed in distilled water. Excess water was blotted prior to the hematoxylin staining. The samples were stained in hematoxylin solution for 3–5 min and differentiated in acid alcohol, then dipped in ammonia solution. The sections were washed in distilled water and rehydrated in descending grades of alcohol, then counterstained in eosin solution for 5 min, and washed in distilled water for 1 min. Per-mount was allowed to spread beneath the coverslip, covering all the tissues. Images were acquired using a microscope (NIKON Eclipse ci, NIKON, Japan) and analyzed with image analysis system (NIKON digital sight DS-FI2, NIKON, Japan).

### Masson’s Trichrome Staining

All heart specimens from the area equidistant to the papillary muscle level between the ligation point and the apical section were subjected to 4% paraformaldehyde-fixed, paraffin-embedded solution. The sections were dewaxed in xylene, rehydrated in descending grades of ethanol, and washed in distilled water. The sections were stained in iron hematoxylin solution for 3 min and differentiated in acid alcohol solution, then washed in distilled water (kits from Beijing G-CLONE Biological Technology Co. Ltd., China, RS3960). The sections were stained in Ponceau acid fuchsin for 5–10 min and rinsed in distilled water. The slides were placed in phosphomolybdic acid solution for 1–3 min then stained in aniline blue solution for 3–6 min. The sections were differentiated in 1% glacial acetic acid and dehydrated in ethanol, followed by xylene for 5 min. Per-mount was allowed to spread beneath the coverslip by covering all the tissues. Images were acquired using the microscope (NIKON ECLIPSE E100, Japan) with image analysis system (NIKON DS-U3, Tokyo, Japan). Fibrosis was analyzed using the Image-pro plus 6.0 software (Media Cybernetics, Inc., MD, USA).

### Whole-Mount Immunohistochemistry

The mice were euthanized and perfused with PBS or fixative. Heart tissues from the area equidistant to the papillary muscle level between the ligation point and the apical section were immersion-fixed in 10% neutral buffered formalin. Tissues were trimmed, processed, embedded, sectioned, and stained for TNF-α (1:200, sc-52746, Santa Cruz Biotechnology, USA), and COL1a (1:1000, GB11022-3, Servicebio, Beijing, China). Goat Polyclonal Secondary Antibody to Rabbit IgG (H&L) was purchased from BioVision (1:1000, 6927–100, CA, USA).

### qPCR

Total RNA was isolated from the area equidistant to the papillary muscle level between the ligation point and the apical section of ischemic heart or the skin of acupoint position using Fast Pure Cell/Tissue Total RNA Isolation Kit (RC101-01, Vazyme, Nanjing, China), and the concentration of isolated RNA was determined with a Qubit RNA BR assay (Invitrogen, Q10211, CA, USA), and cDNA was prepared using HiScript Q RT Super Mix for qPCR (+ gDNA wiper) (R123-01, Vazyme, Nanjing, China) according to the manufacturer’s instructions. The mRNA levels were assessed on an ABI QuantStudio6 Q6 Real-time PCR system (ABI, USA) by qPCR using ChamQ Universal SYBR qPCR Master Mix (Q711-02, Vazyme, Nanjing, China). The relative expression of mRNA was calculated by △△Ct according to standard methods. The primer sequences were as follows: *Actin*, forward: 5′-CTCTCCCTCACGCCATC-3′, reverse: 5′-ACGCACGATTTCCCTCTC-3′; *P*2 × 7, forward: 5′-GCCTGAGCTACATCGCA-3′, reverse: 5′-GGGGTCCAACACTCTCTTC-3′; *Serca*, forward: 5′-GAGAACGCTCACACAAAGACC-3′, reverse: 5′-ACTGCTCAATCACAAGTTCCAG-3′; *Cav*1.2, forward: 5′-GGTGGGGAGTGAGGAAA-3′, reverse: 5′-CAAGGCTGGCAACAGAG-3′; *Col*1a, forward: 5′-CAGAGGCGAAGGCAACA-3′, reverse: 5′-GTCCAAGGGAGCCACATC-3′; *Tnf*-α, forward: 5′-CGCTGAGGTCAATCTGC-3′, reverse: 5′-GGCTGGGTAGAGAATGGA-3′; *Il*6, forward: 5′-GCCTTCTTGGGACTGATGCT-3′, reverse: 5′-TGCCATTGCACAACTCTTTTC-3′; *Cthrc*1, forward: 5′-CCATCGAAGCCATCATCT-3′, reverse: 5′-TACCAATCCAGCACCAATC-3′; *Il*5-α, forward: 5′-AGGCTTCCTGTCCCTACTCAT-3′, reverse: 5′-CCTCGCCACACTTCTCTTTTTG-3′; *Wfdc*12, forward: 5′- CCTCGCCACACTTCTCTTTTTG-3′, reverse: 5′-CCTCGCCACACTTCTCTTTTTG-3′; *Wnt*4, forward: 5′-CGAGCAATTGGCTGTACCTG-3′, reverse: 5′-GGGAGTCCAGTGTGGAACAG-3′; *Doc*2b, forward: 5′-ACTGGCTGATCCCTACGTCA-3′, reverse: 5′-ACTGGCTGATCCCTACGTCA-3′; *Erdr*1, forward: 5′-ACAAGGTAGGAAGCCTGCG-3′, reverse: 5′-CGGTGGACGCTGACG-3′.

### RNA-seq and Computational Analysis for RNA-seq Data

RNA was extracted from the skin of the acupoint location at PC6, BL15, and ST36 of mice in each group and the library was prepared according to the TruSeq RNA Sample Preparation v2 (Illumina, 15,025,062) protocol. cBot Multiplex re-hybridization plate and TruSeq SBS kit V3 (Illumina, 15,021,668) were used for cluster generation. Sequencing was performed using Illumina HiSeq 2500 (Illumina, USA). According to the generated database, differentially expressed genes were filtered by Log2 fold change (FC) ≥|± 1|FDR < 0.05, followed by GO analysis, KEGG enrichment (for specific methods, please refer to previous studies [4, 19, 20]).

### Statistical Analysis

All data are presented as mean ± SD. Statistical analysis was done using one-way analysis of variance (ANOVA) with the Turkey test using GraphPad Prism 7.0 (GraphPad Software Inc., La Jolla, CA, USA) and SPSS 20.0 software (IBM, Chicago, USA). A value of *P* < 0.05 was considered statistically significant.

## Results

### Comparison of Mouse Mortality, Incidence of Arrhythmia, and Electrophysiology Among the Three Acupoints Group

After 7 days of acupuncture intervention, the mice survival rate was 88.9% in the PC6 group, 80% in the BL15 group, and 100% in the ST36 group (Supplementary Table 1). Spontaneous premature ventricular complexes (PVCs) were not observed in the Sham group (0%) but emerged in the MI group (28.6%). Incidence of arrhythmia in the PC6 group was reduced to 11.1%, while the rate increased to 50% in the BL15 group, and changed (25%) in the ST36 group (Supplementary Table 1). These results indicate that PC6 is the safest acupoint for MI treatment compared to the other two acupoints. Although the BL15 acupoint reduced mortality, it increased arrhythmias. The ST36 acupoint did not alter the arrhythmia rate, but less effective.

**Fig. 1:**
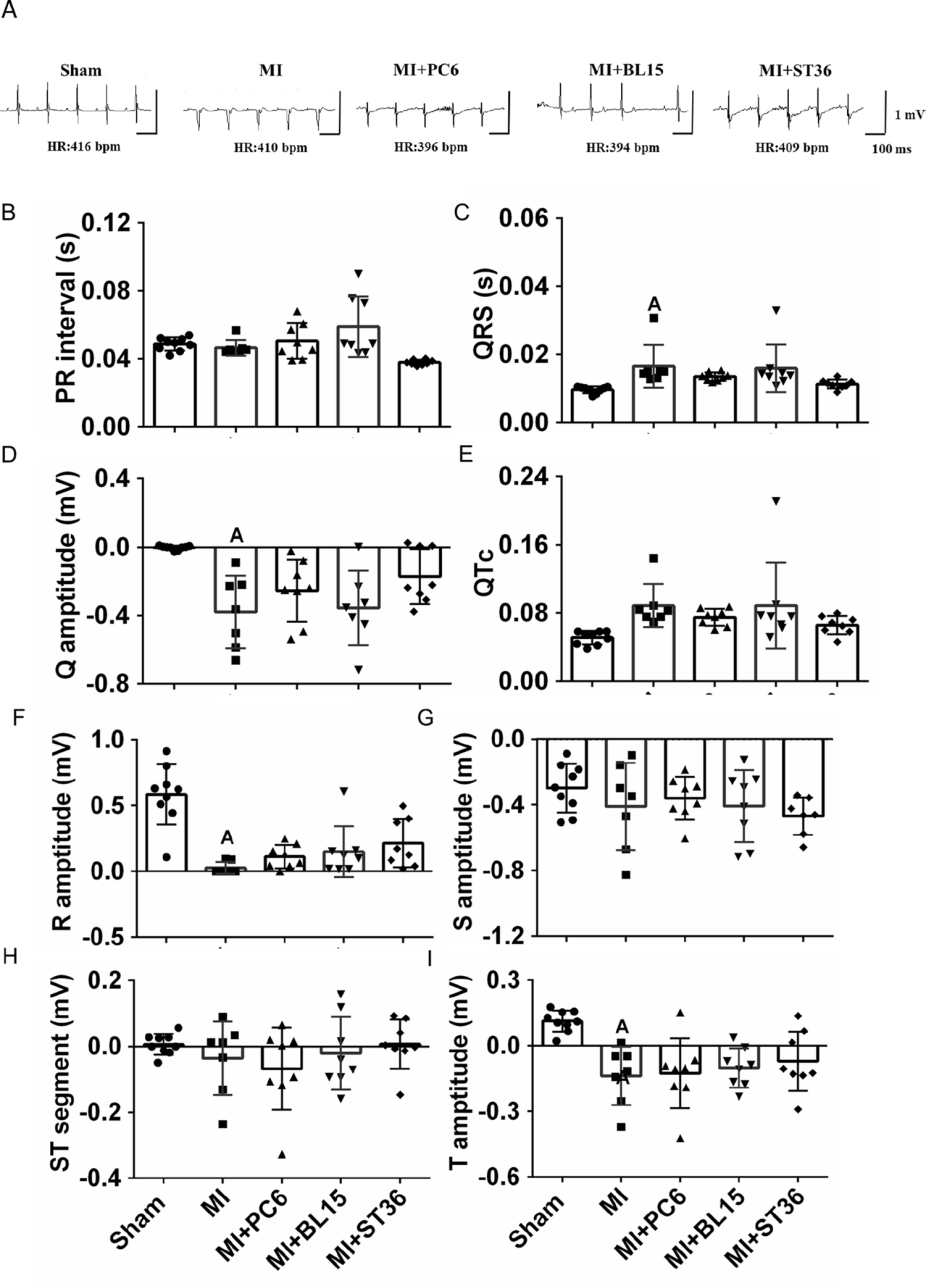

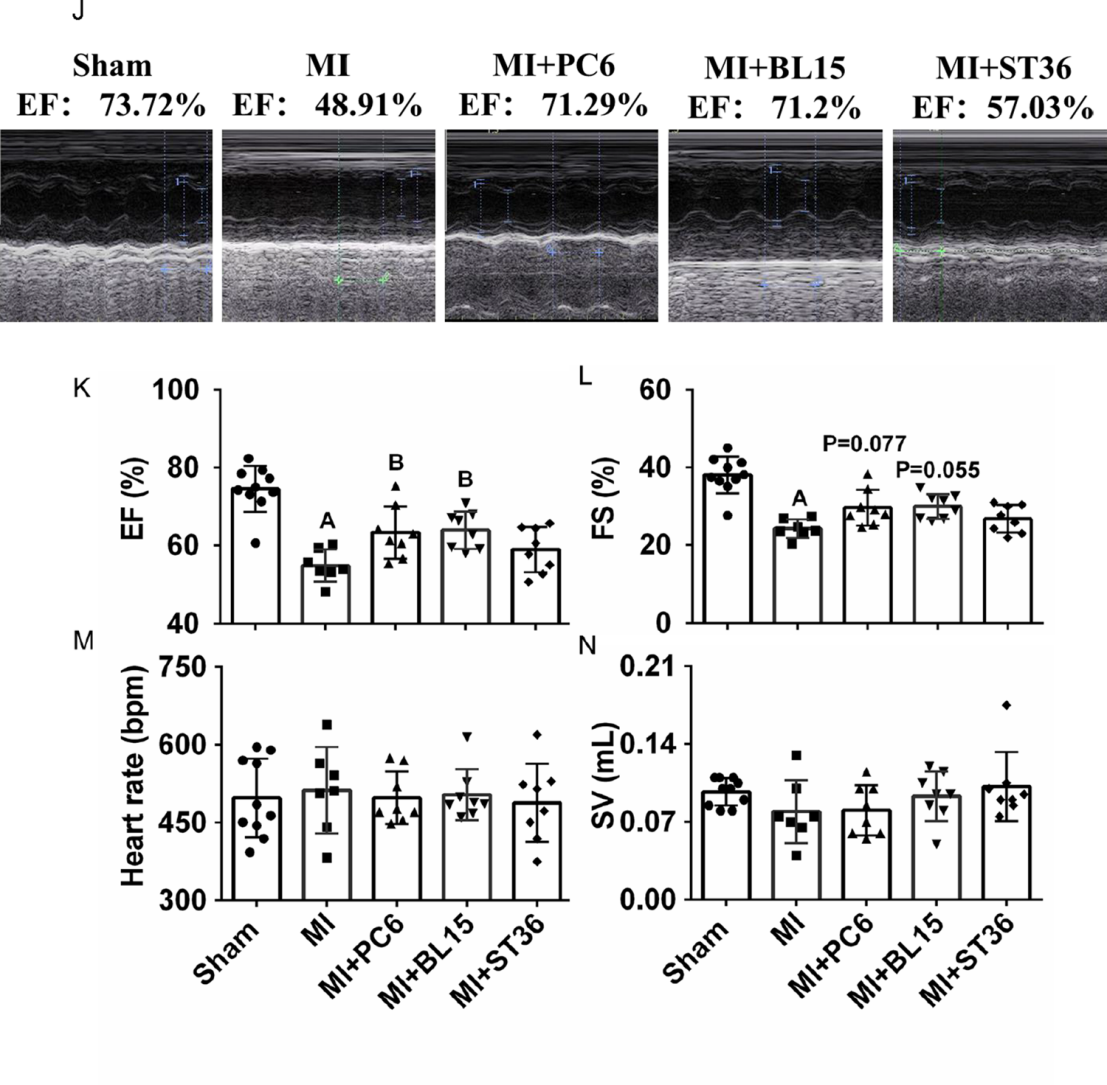
Electrophysiology and cardiac function in the Sham, MI, MI + PC6, MI + BL15, and MI + ST36 groups. **A** Representative electrocardiogram recordings in the Sham, MI, MI + PC6, MI + BL15, and MI + ST36 groups. **B**–**I** Summary of the electrophysiology, including the PR interval, QRS width, QTc, Q amplitude, R amplitude, S amplitude, ST segment, and T amplitude in the Sham (*n* = 9), MI (*n* = 7), MI + PC6 (*n* = 8) groups, MI + BL15 (*n* = 8), and MI + ST36 (*n* = 8) groups. **J** Representative echocardiograms from parasternal short-axis images at the midpapillary level in the Sham (*n* = 9), MI (*n* = 7), MI + PC6 (*n* = 8), MI + BL15 (*n* = 8), and MI + ST36 (*n* = 8) groups. A summary of echocardiographic parameters is listed below (**K**–**N**). **K** Ejection fraction (EF). **L** Fractional shortening (FS). **M** Heart rate (HR). **N** Stroke volume (SV). A represents *p* < 0.05 vs. the Sham group, B represents *p* < 0.05 vs. the MI group

The typical electrocardiogram traces are shown in Fig. 1A. Compared with the Sham group, QRS width and Q amplitude were significantly increased, while R amplitude was reduced in the MI group. No significant difference was detected on the ECG parameters after 7 days of intervention by either PC6, BL15, or ST36 treatment (Fig. 1B–I).

### Acupuncture at PC6 and BL15 Improved Cardiac Function and Reduced Fibrosis

Typical echocardiogram traces for the effects of PC6, BL15, and ST36 were used to evaluate cardiac function and structure (Fig. 1J). Ejection fraction (EF) and fraction shortening (FS) in the MI group were significantly reduced compared to those in the Sham group. Both PC6 and BL15 intervention reversed the reduction of EF effectively, but ST36 could not restore EF (Fig. 1K). FS showed no significant change among these three acupoints, while the same was observed in stroke volume (SV) and heart rate (HR) among the five groups (Fig. 1L–N).

**Fig. 2:**
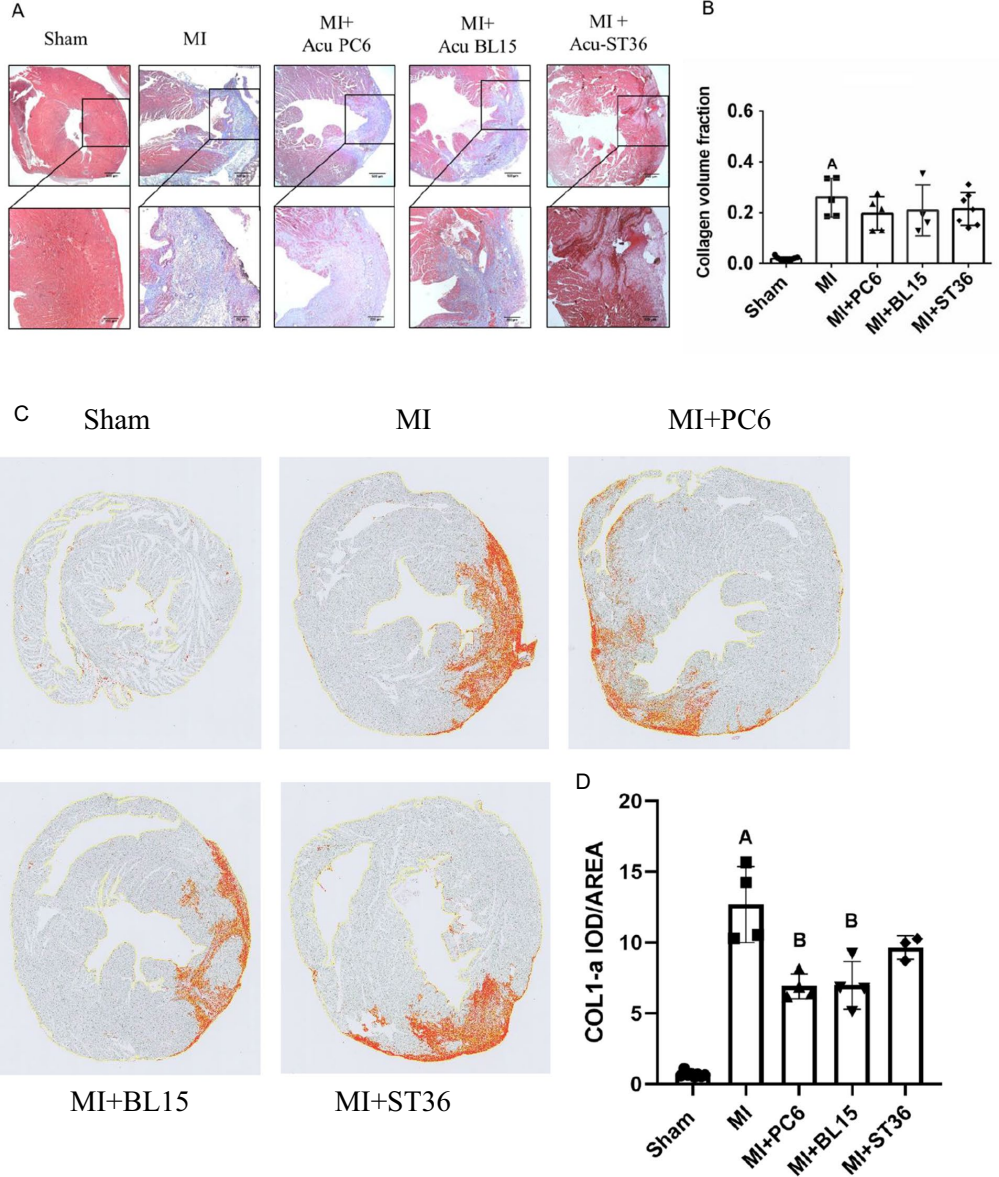

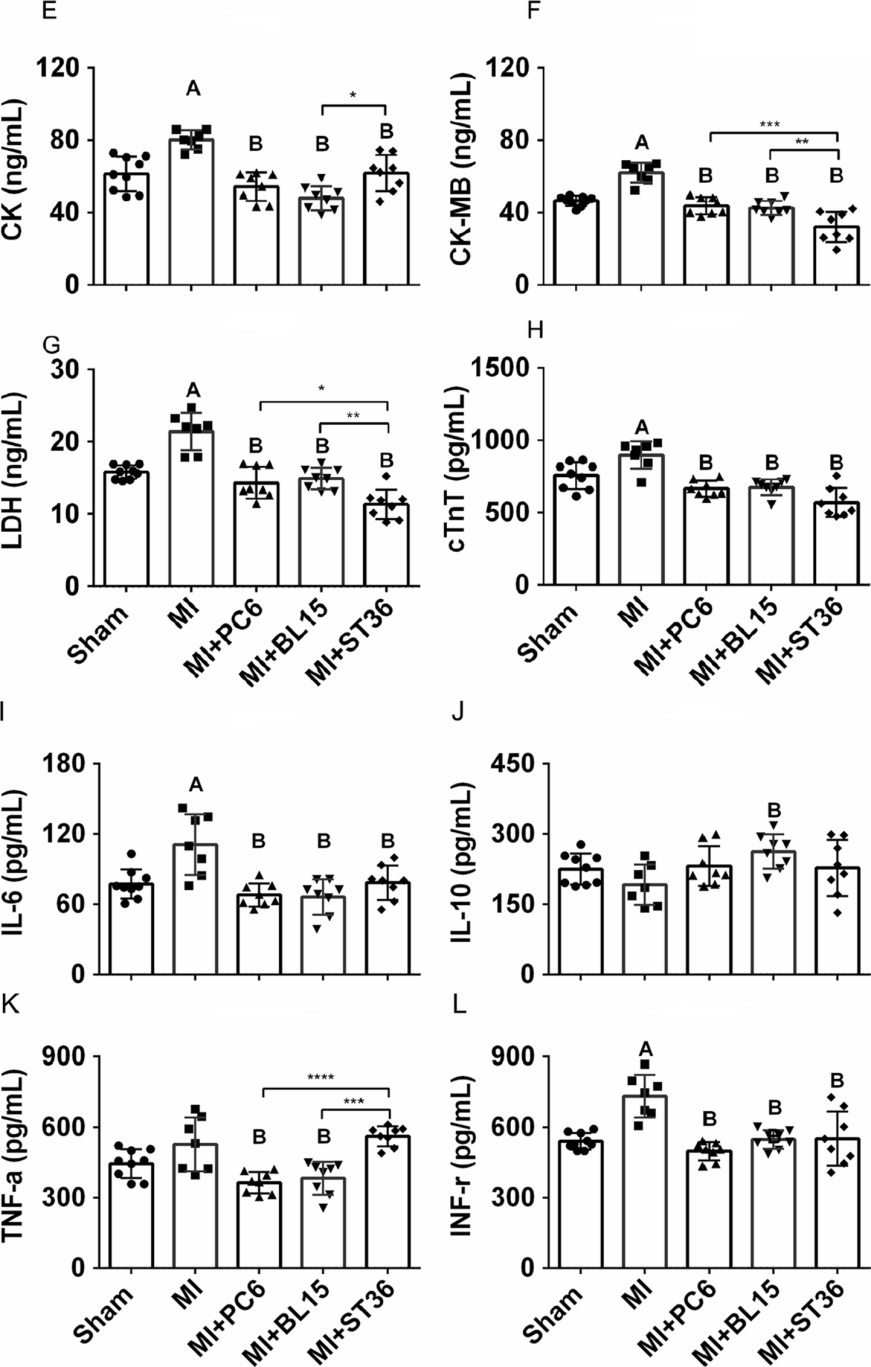

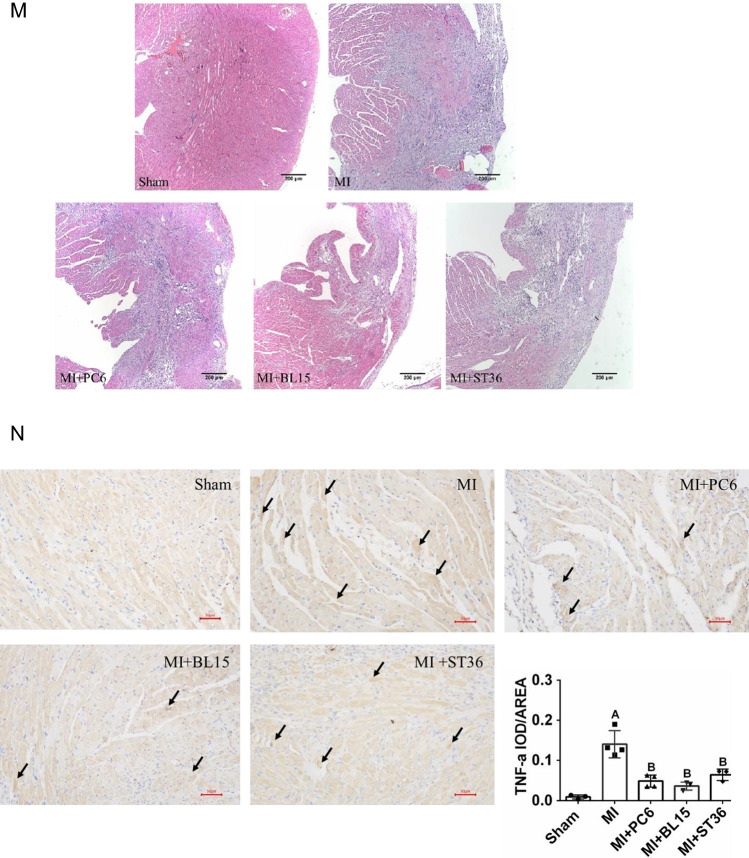
Acupuncture reduced fibrosis, improved myocardial enzyme and anti-inflammatory in heart and serum. **A** Tissue sections and fibrosis in the ventricle determined by calculating collagen deposition after Masson’s trichrome staining in the Sham, MI, MI + PC6, MI + BL15, and MI + ST36 group mice. Scale bar: 200 μm. **B** Collagen deposition index in these groups, *n* = 3 in each group. **C**–**D** Representative COL1-α immunohistochemical staining and analysis of heart tissues in the Sham, MI, MI + PC6, MI + BL15, and MI + ST36 groups, *n* = 3 in each group. **E** The serum CK level. **F** The serum CK-MB level. **G** The serum LDH level. **H** The serum cTnT level in the Sham, MI, MI + PC6, MI + BL15, and MI + ST36 groups. *n* = 7 ~ 9 in each group. **I** The serum IL-6 level. **J** The serum IL-10 level. **K** The serum TNF-α level. **L** the serum INF-γ level, in the Sham, MI, MI + PC6, MI + BL15, and MI + ST36 groups, *n* = 7 ~ 9 in each group. **M** Hematoxylin and eosin staining (HE) staining of heart tissue, *n* = 3 in each group. **N** Representative TNF-α immunohistochemical staining and analysis of heart tissues in the Sham, MI, MI + PC6, MI + BL15, and MI + ST36 groups, *n* = 3 in each group. A represents *p* < 0.05 vs. the Sham group, B represents *p* < 0.05 vs. the MI group

The collagen deposition index in the ventricle was detected by Masson’s trichrome staining, which showed fibrosis levels using red and blue colors. Fibrosis increased in the MI group, compared with the Sham group. Acupuncture at PC6, BL15, and ST36 significantly reduced the degree of fibrosis (Fig. 2A–B). Immunohistochemistry was implemented to detect the local collagen deposition of the heart furtherly. The result indicated that acupuncture at PC6 and BL15 lowered the COL1a, a fibrotic marker, whereas ST36 did not have any effect (Fig. 2C–D, see blue arrow indicates positive COL1a expression area). Myocardial enzymology was examined to assess the extent of cardiac damage. Serum CK, CK-MB, LDH, and cTnT increased in the MI group, whereas PC6, BL15, and ST36 acupuncture lowered these parameters effectively (Fig. 2E–H).

### Acupuncture at Each of Three Acupoints Alleviated the Inflammatory Response Induced by MI

Plasma levels of IL-6 and INF-γ detected by Elisa assay were increased in the MI group compared with those in the Sham group, but they were all decreased in the MI + PC6, MI + BL15, and MI + ST36 group (Fig. 2I, L). TNF-α and renin levels did not show significant difference between the Sham group and the MI group, but were decreased by acupuncture treatment at PC6 and BL15; whereas treatment at ST36 hardly affected this (Fig. 2K, s-Fig. 2). Inflammatory cell infiltration in the ventricles were increased in the MI group compared to the Sham group; however, they were not reversed by acupuncture treatment at PC6, BL15, or ST36 (Fig. 2M). To further detect the inflammatory response, TNF-α level was detected in the local part of the ventricle by immunohistochemical staining. Compared with the Sham group, TNF-α expression increased sharply in the MI group, while acupuncture treatment at all three acupoints could reduce it effectively (Fig. 2N–O, see black arrow indicates positive TNF-α expression area).

### Expression of Representative Genes of Inflammation, Myocardial Contraction, and Fibrosis After Different Acupoint Stimulation

**Fig. 3:**
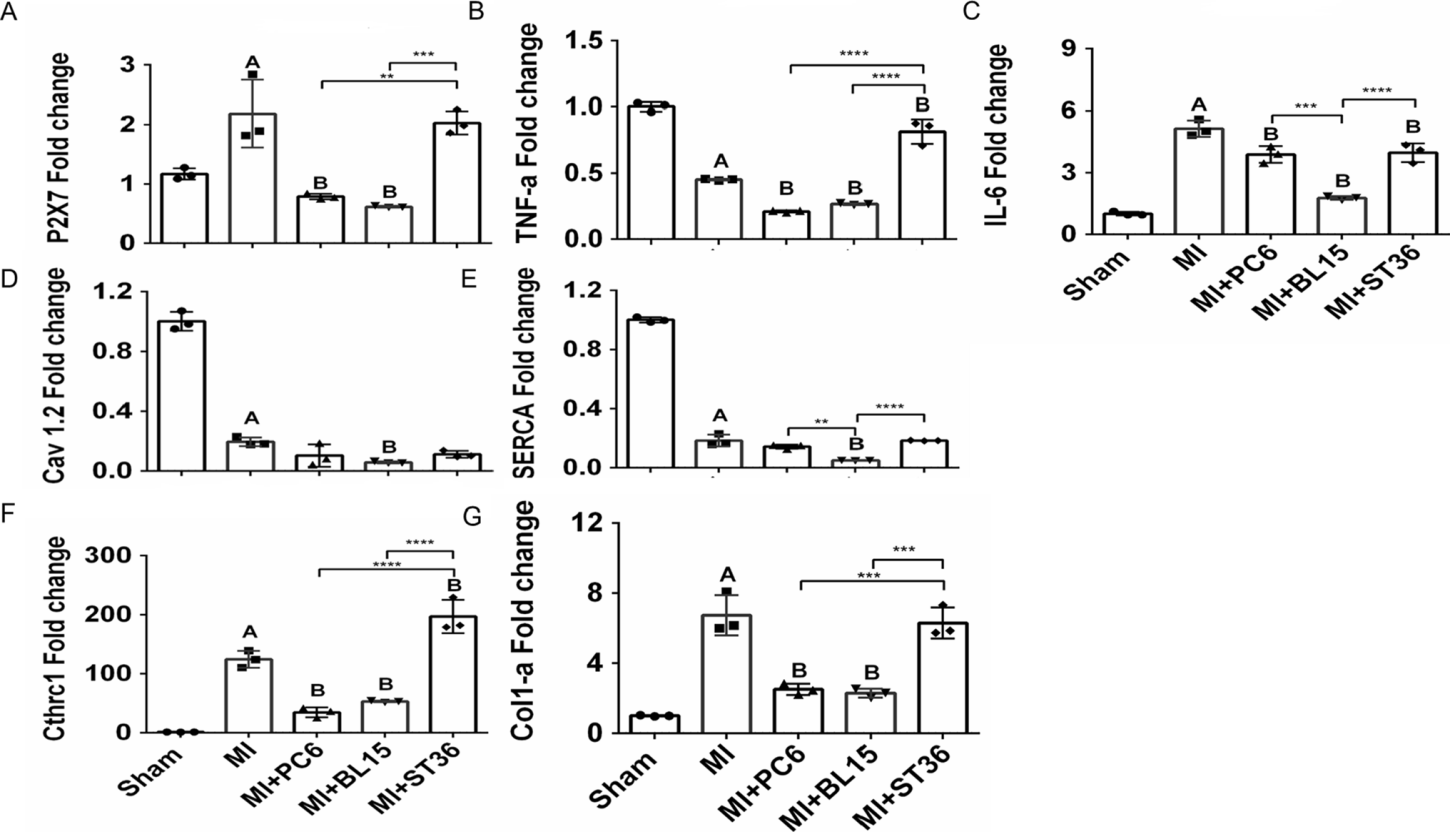
Expression of representative genes of inflammation, myocardial contraction and fibrosis after different acupoint stimulation. **A**–**C** *P2* × *7*, *Tnf-α*, *Il-6* mRNA expression in the heart. **D**–**E** *Cav 1.2*, *Serca* mRNA expression in the heart. **F**–**G** *Cthrc1*, *Col1-α* mRNA expression in the heart. A represents *p* < 0.05 vs. the Sham group, B represents *p* < 0.05 vs. the MI group, *n* = 3 in each group

We further verified the mRNA level of some representative genes of inflammation, such as *P2X7*, *IL-6*, and *TNF-α* by qPCR. The expressions of *P2X7* and *IL-6* were increased in the MI group, but decreased in the MI + PC6, MI + BL15, or MI + ST36 group. However, ST36 increased the *TNF-α* level when compared with the MI group, while PC6 and BL15 reduced it (Fig. 3A–C). *Serca* and *Cav1.2* are typical markers of calcium channels which affect myocardial contraction. They decreased sharply after MI operation. BL15, but not PC6 and ST36 treatment, lowered the expression of *Serca* and *Cav1.2*, even lower than the Sham group. This result partly explained why BL15 treatment caused arrhythmias when it was applied to treat MI injury during the acute phase (Fig. 3D–E). qPCR further demonstrated that acupuncture at PC6 and BL15 reduced the fibrotic markers of *Col1a* and *Cthrc1*, but ST36 exhibited a promotion effect on them (Fig. 3F–G).

### Acupuncture at Three Acupoints Altered Gene Expression Profiles Differently in the Skin Tissue of Local Acupoints

To understand the differences in the local tissue where the PC6, BL15, or ST36 acupoints are located and the link to the phenotypes in the heart, we conducted gene expression profiling for the three acupoints by using next-generation high-throughput sequencing (RNA-seq analysis). Compared to the Sham group, 299 genes were differentially expressed in the tissue of the PC6 acupoint area when the heart was under ischemic conditions. Of these 299 genes, 242 (80.9%) were upregulated, and 57 (19.1%) were downregulated. Differentially expressed genes in the MI + BL15 and MI + ST36 groups were less than the MI + PC6 group; only 65 genes in the MI + BL15 group (including 56 genes unregulated [86.2%], and 9 genes downregulated [13.8%]) and 6 genes in the MI + ST36 group (4 genes upregulated and 2 genes downregulated), indicating a correlation between myocardial ischemic disease and meridian specificity-related acupoints. After acupuncture treatment, DEGs increased in all three acupoint groups. There was also no doubt that the number in the MI + PC6 group (1070) was still the most, compared to the MI + BL15 (900) and MI + ST36 (292) groups. Details were listed in Table 1.

**Table 1:** Differentially expressed genes with a log2(FC) >|± 1| and *p* value < 0.05

DEGs	MI vs Sham			Acu vs MI		
	PC6	BL15	ST36	PC6	BL15	ST36
Up	**242 (80.9%)**	**56 (86.2%)**	**4 (66.7%)**	**561 (52.4%)**	**536 (59.6%)**	**192 (65.8%)**
Down	**57 (19.1%)**	**9 (13.8%)**	**2 (33.3%)**	**509 (47.6%)**	**364 (40.4%)**	**100 (34.2%)**
Total	**299 (100%)**	**65 (100%)**	**6 (100%)**	**1070 (100%)**	**900 (100%)**	**292 (100%)**

*DEGs*, differentially expressed genes; *MI*, myocardial infarction; *Acu*, acupuncture; PC6, Neiguan; BL15, Xinshu; ST36, Zusanli

**Fig. 4:**
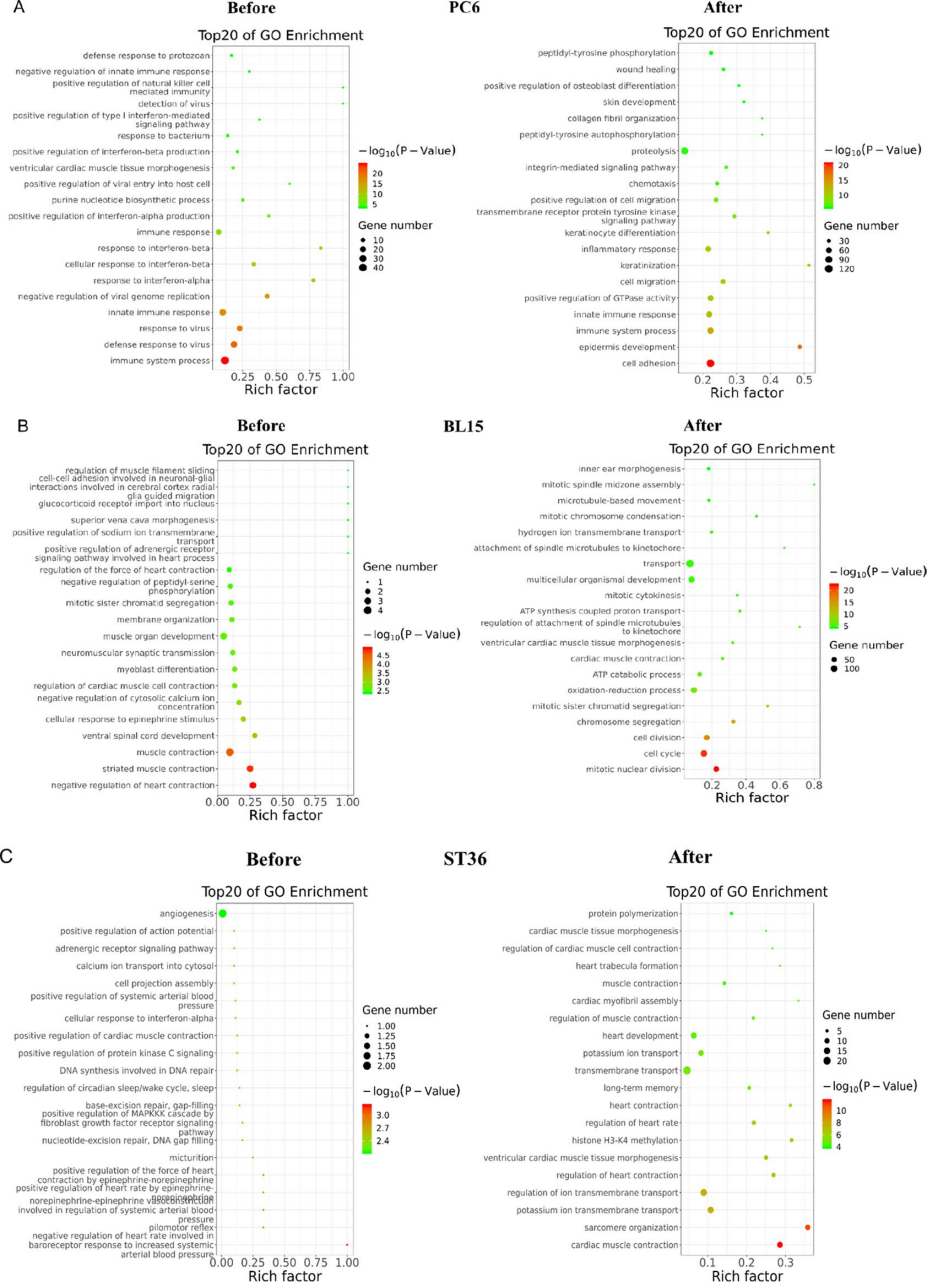

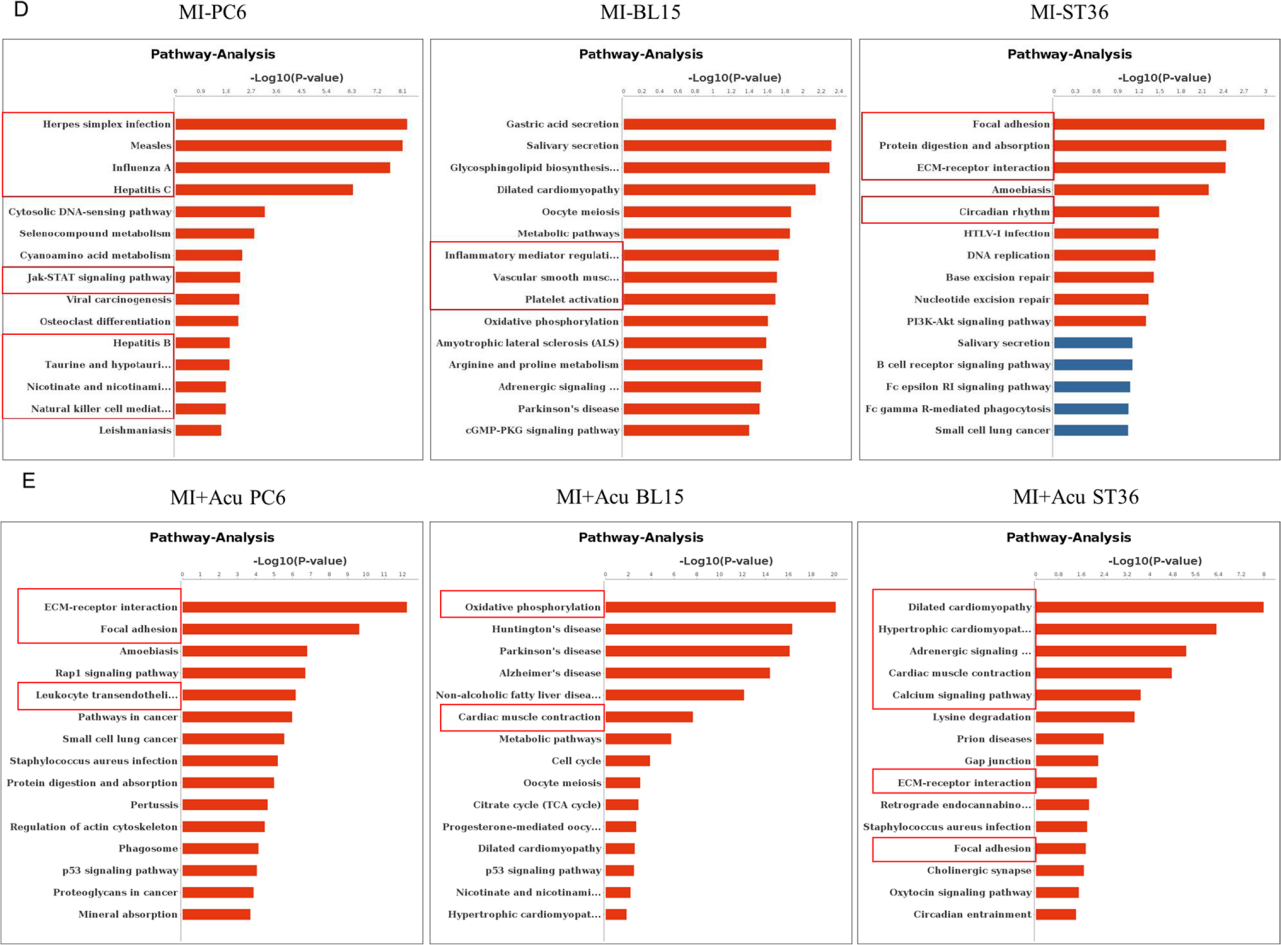
Differentially expressed genes (DEGs) altered in acupoints by acupuncture treatment. **A** Top 20 Go ontology (GO) analysis of the DEGs altered in PC6 acupoint before and after acupuncture. **B** Top 20 GO analysis of the DEGs altered in BL15 acupoint before and after acupuncture. **C** Top 20 GO analysis of the DEGs altered in ST36 acupoint before and after acupuncture treatment. **D**, **E** Top 15 KEGG pathways of the DEGs altered in PC6, BL15, and ST36 acupoint before and after acupuncture treatment separately, *n* = 3 in each group

Gene ontology (GO), including biological process (BP), cellular component (CC), and molecular function (MF) annotation, indicated that the co-regulated genes in the acupoints displayed different patterns among these three acupoints. Most of the top 20 GO terms before and after acupuncture in the MI + PC6 group were related to immune function, including immune response, response to interferon-β, innate immune response, immune system process, and response to bacterium (Fig. 4A). In the MI + BL15 group, many muscle contraction processes were involved before and after acupuncture treatment, including regulation of the force of heart contraction, cardiac muscle contraction, positive regulation of adrenergic receptor, striated muscle contraction, and ATP synthesis and catabolic process; those processes were possibly related to the highest arrhythmia incidence and lowest survival rate in this group (Fig. 4B). In the MI + ST36 group, only some genes correlated to iron, transmembrane transport, and regulation of heart rate processes were included (Fig. 4C). Kyoto Encyclopedia of Genes and Genomes (KEGG) pathway analysis from DAVID confirmed that these genes belonged to the same functional pathways as the biological process and molecular function displayed in GO analysis of these three groups. The most prominent signaling was the immune-virus correlation pathway in the MI + PC6 group, like influenza A, hepatitis C, natural killer cell mediated cytotoxicity, and leukocyte transendothelial migration; while in the MI + BL15 group, cardiac muscle, vascular smooth muscle contraction, adrenergic signaling in cardiomyocytes, and platelet activation were observed. As the number of DEGs was too small in the MI + ST36 group, enriched pathway was not analyzed in this study (Fig. 4D–E).

**Fig. 5:**
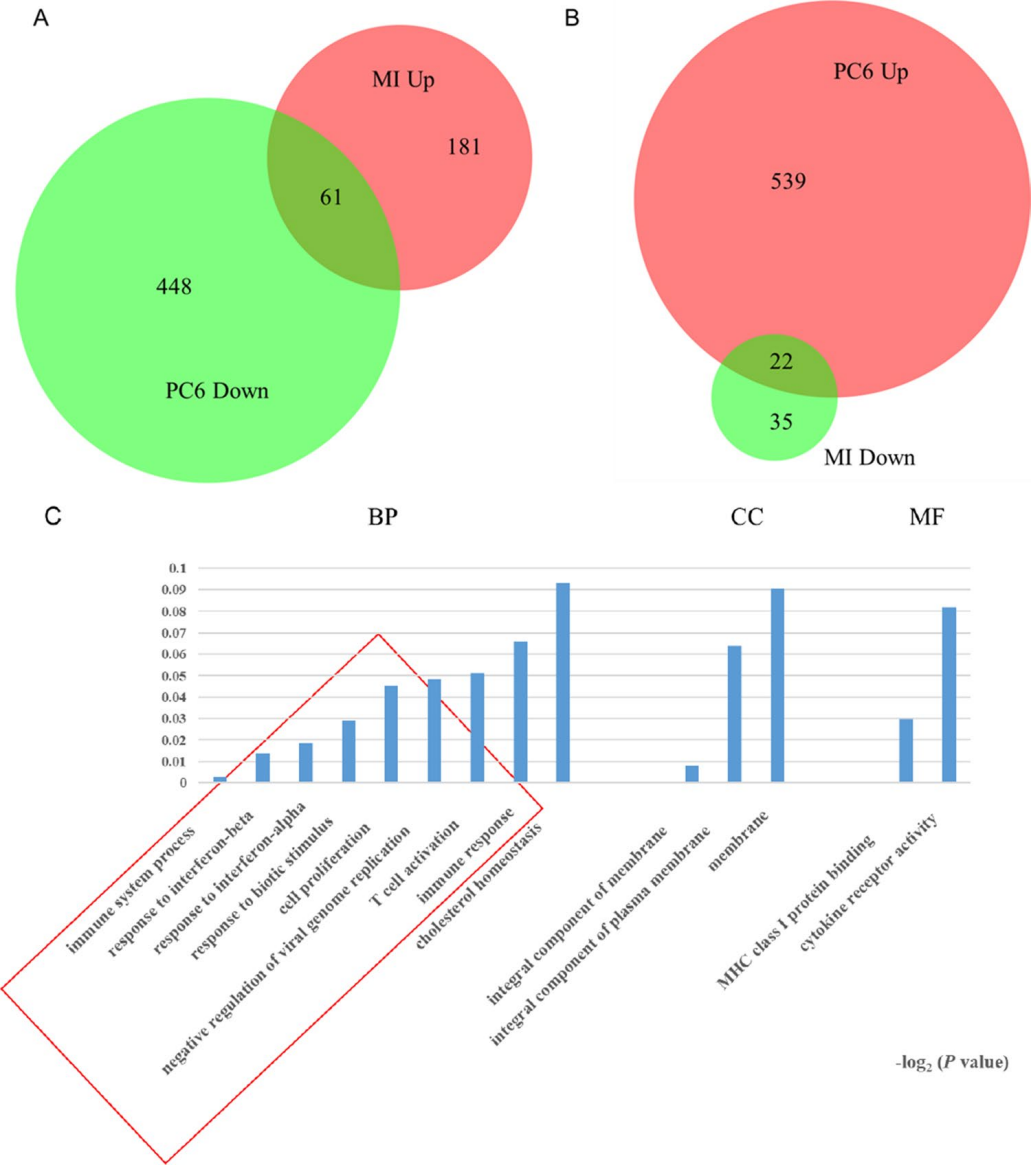

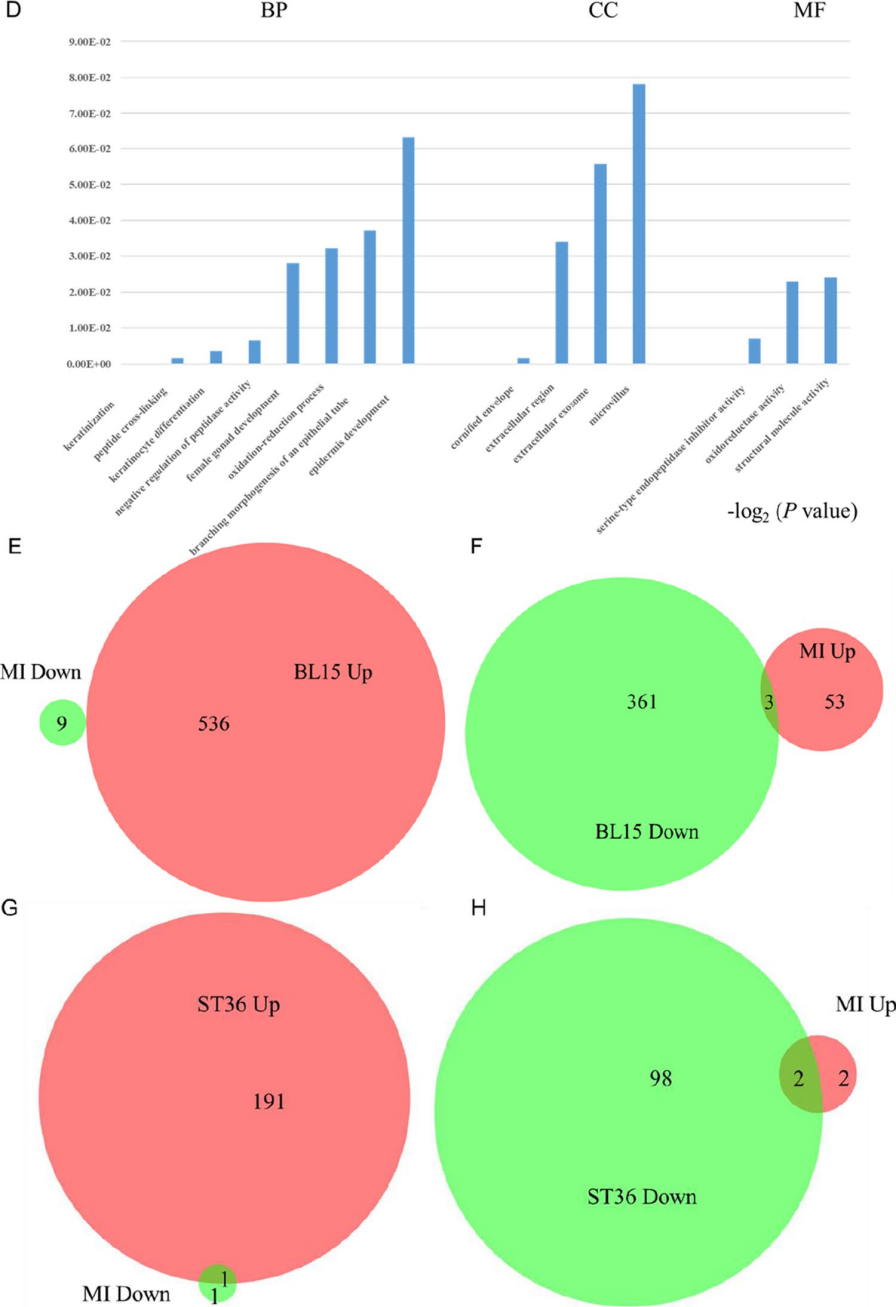

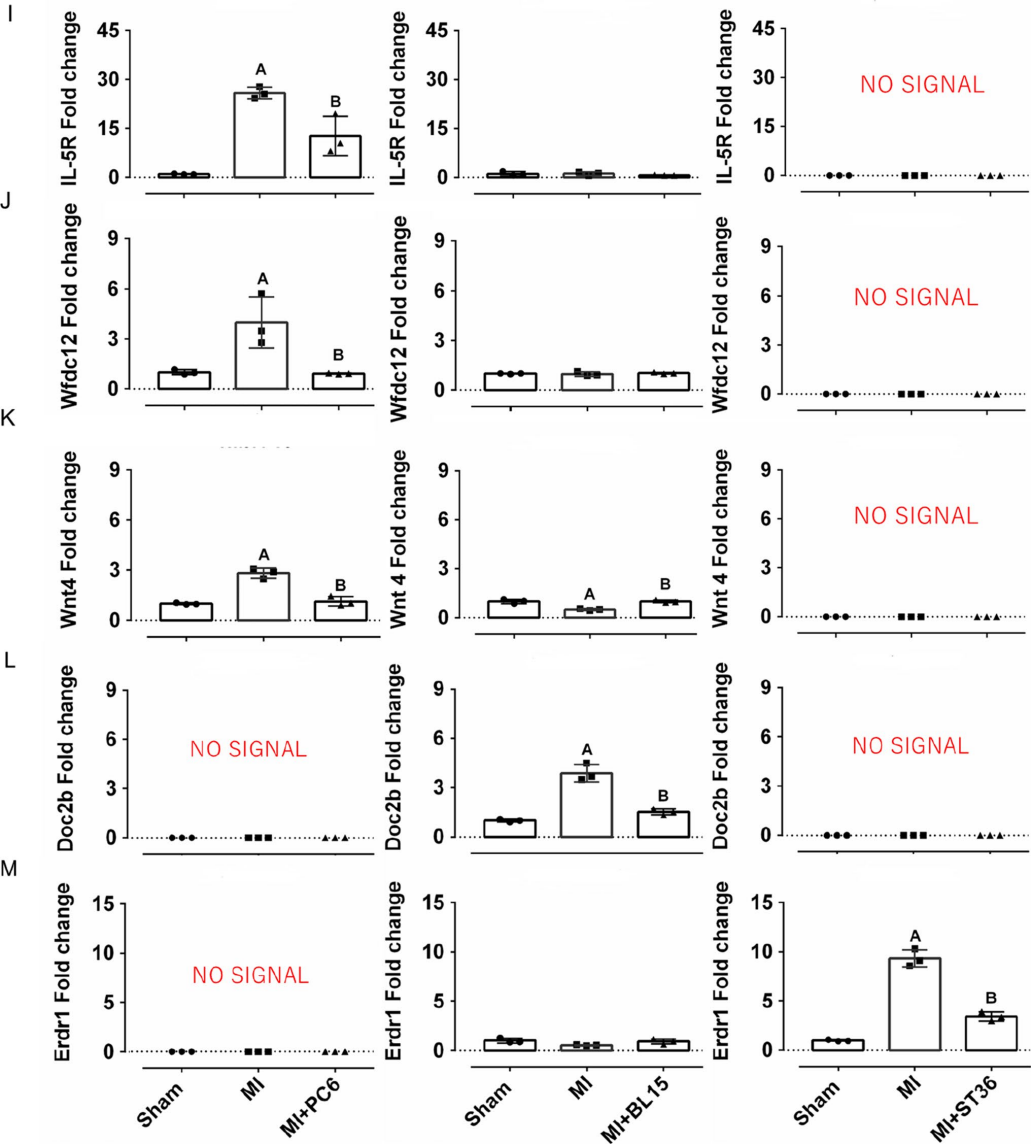
Further analysis of unique genes in the MI + PC6, MI + BL15, and MI + ST36 groups and qPCR verification of some unique genes for different acupoints. **A**, **B** Venn diagram of the DEGs in PC6 acupoint before and after acupuncture. **C**, **D** GO analysis of the co-regulated genes in the PC6 acupoint before and after acupuncture. **E**, **F** Venn diagram of the DEGs in BL15 acupoint before and after acupuncture. **G**, **H** Venn diagram of the DEGs in ST36 acupoint before and after acupuncture. There is no GO analysis both in BL15 acupoint and ST36 acupoint due to very few co-regulated genes exist. **I**–**M** *Il-5R*, *Wfdc12*, *Wnt4*, *Doc2b*, *Erdr1* mRNA expression in the acupoints. Some acupoints unique genes displayed no signal in the qPCR assay. Panel **A** represents *p* < 0.05 vs. the Sham group. Panel **B** represents *p* < 0.05 vs. the MI group, *n* = 3 in each group

To explore the potential mechanism of acupuncture treatment based on acupoint specificity, we compared the gene expression profiles of MI mice with that of different acupuncture treated mice. Further analysis of the genetic changes unique to each acupoint were assessed carefully. In the upregulated genes (242) of PC6 treatment after MI, only 61 genes were reversed in the MI + PC6 group, while acupuncture could regulate the other 448 genes which were unchanged in the MI group. Furthermore, in the downregulated genes (57) of PC6 treatment after MI, about 38.6% genes (22/57) were upregulated by acupuncture, while it could upregulate the other 539 genes which did not appear in the MI group (Fig. 5A–B). GO analysis of these co-regulated genes (especially the 61 genes) displayed that many immune function processes were involved, such as immune system process/response, response to interferon α/β, biotic stimulus, negative regulation of viral genome, and T cell activation (Fig. 5C–D). For the BL15 and ST36 group, the number of co-regulated genes were much smaller than that in the PC6 group (Fig. 5E–H), reflecting the specificity of diseases and acupoints to some extent. Moreover, to verify the RNA-Seq data, we specifically checked some unique genes enriched in the three groups uniquely using qPCR. Genes related to inflammation in the PC6 group (*IL-5r*, *Wfdc12*, *Wnt4*), ion channel in the BL15 group (*Doc2b*), and fibrosis in the ST36 group (*Erdr1*) were detected. These unique genes were changed only in their respective groups and displayed no significant change or no signal detected of qPCR assay in the other two groups (Fig. 5I–M).

## Discussion

The existence of acupoint and meridian specificity is controversial [21]. Experimental studies are needed to elucidate the point-specific actions of acupuncture. The current study focuses on the acupoint specificity from multiple dimensions’ evaluation in acupuncture treatment on mice MI model using different acupoints (PC6, BL15, and ST36). Firstly, we found that the changes in electrophysiology was not obvious among these three groups after 7 days of acupuncture treatment, while they all could improve the myocardial enzyme spectrum and decrease the level of inflammatory factors both in the serum and local myocardial tissue. We then observed that PC6 and BL15 were superior to ST36 in improving cardiac function. Renin is a proteolytic enzyme released by the paraglular cells of the glomerular apparatus, which are innervated by sympathetic nervous. A variety of causes including myocardial infarction can increase renin release [22]. In our study, renin level in the serum did not increase sharply 7 days after surgery, but acupuncture could decrease it effectively. These changes could be the generality characteristics among the three acupoints.

Next, we detected respective characteristics from the level of gene expression. The huge number of altered DEGs and pathways related to immune system in local acupoint may partially explain the efficacy of PC6. We found that BL15 treatment cause a high incidence of arrhythmia, which did not show in the PC6 and ST36 groups. Ion channel-related genes from the RNA-seq analysis were altered by BL15 treatment, supporting the observations. Combing higher efficacy, safety issues, and RNA-seq results, PC6 holds point specificity in treating heart diseases and improved safety compared to BL15 and ST36. We confirmed that PC6 is the safest and effective acupoint for ameliorating symptoms after MI.

Numerous studies have demonstrated the effectiveness of acupuncture in multiple pathways and targets, and have tried to reveal the mechanisms from local acupoints to target organs (review) [23]. As believed traditionally, acupuncture meridians constitute channels connecting the surface of the body to internal organs [24], and acupoints possess a three-dimensional structure, which are encased in a dense distribution of nerves, regular arrangement of blood vessels, and rich inherent connective tissue under a thin layer of skin [25]. Acupuncture can enhance the network communication among many cells, signal transduction, and molecules in acupoints, which mediate the therapeutic effects and transmit acupuncture information and energy from the acupoints to target organs [26, 27].

PC6 is the most frequently used acupoint for heart disease in animal studies and clinical research. It has been demonstrated that acupuncture at PC6 can ameliorate inflammation [9], inhibit collagen deposition and myocardial hypertrophy [28], modulate autonomic nerve balance [29], and regulate endothelial cell function [30]. Long-term acupuncture at PC6 can also suppress premature ventricular complexes (PVCs) occurring post-myocardial ischemia by alleviating inflammation and fibrosis [6]. In this study, we detected IL6, IL10, TNF-α, and INF-γ level in the serum and COL1α expression in the ischemic heart to assess inflammation and fibrosis degree. We found that acupuncture at PC6 could reverse the elevated IL6, TNF-α, and INF-γ levels in the serum and TNF-α expression in the heart. The abnormal accumulation of collagen fibers was also reduced due to the inhibition of COL1α expression partly. These functional changes are closely related to subsequent changes in a large number of differentially expressed genes (DEGs). Many genes and signal pathways related to inflammation are involved, which is consistent with other studies. It has been reported that the anti-inflammatory actions and mechanisms of acupuncture from acupoint to target organs is closely linked to neuro-immune regulation [21]. Because PC6 is located at the pericardial meridian and far away from the heart, which directly belong to heart divergent meridian, it is the most effective and safest acupoint, when compared to BL15 and ST36. Future studies are required to elucidate the anti-inflammatory mechanisms of acupuncture from PC6 to heart via the neuro-immune regulation.

BL15 is located approximately 1.5 cm lateral to the lower border of the spinous process of the fifth thoracic vertebra, which is innervated by vagus and some T5 sympathetic nerves [31]. With a promotion of systolic function, acupuncture at BL15 can improve myocardial enzyme profiles and also reduce inflammation and fibrosis in the heart. Although BL15 can improve systolic function, a high incidence of arrhythmia limited its application. Given the safety and effectiveness, it was not as safety as PC6. The stimulation of BL15 with acupuncture might affect the conduction of sympathetic nerves of plexus cardiacus, thus altering the heart rate and causing arrhythmia easily [32, 33], which is the most serious complication after myocardial ischemia [34].

ST36 is frequently used to treat gastrointestinal diseases other than cardiovascular diseases according to literature analysis [35]. In addition, it is always used to help healthy “*Qi*” (energy) and improve the body’s immunity [36], which is often combined with other acupoints when treating diseases [37–39]. Our study confirmed that ST36 improved the systolic function without statistical significance, which the extent was not as greater as PC6 and BL15 treatment. Although the effect of improving cardiac function was not very obvious as PC6 and BL15, it could also lower the myocardial enzyme and inflammatory factors. Thus, it is better to use ST36 company with other acupoints to enhance the therapeutic effect, not only based on the traditional Chinese Medicine theory but also rooted in the scientific experiment proof.

We concentrated on the change in gene expression at acupoints themselves with or without acupuncture treatment, which was different from previous studies that focused on changes of gene expression profile in cardiac tissue. The number of the DEGs was much larger in PC6 than in BL15 and ST36, which may support the definition of acupoint sensitization that acupoints will be activated from silence when diseases strike and explain why PC6 is most frequently used in improving symptoms of angina, palpitation, and other heart diseases. Furthermore, GO analysis of these three acupoints also showed specificity in our study. BP, CC, and MF process of PC6 acupoint mainly included immune response, which may correspond to the lower level of fibrosis and inflammation. The muscle contraction process was involved in the BL15 treatment, which is related to the best systolic function in comparison with PC6 and ST36. In addition, ion was altered by BL15 which may link to the high incidence of arrhythmia. Only some ion, transmembrane transport, and regulation of heart rate processes were showed in the MI + ST36 group. These differences explain the specificity of acupoints for a certain disease. More experimental evidence is needed to elucidate the detail mechanism of acupoints specificity in the future.

## Conclusions

**Fig. 6:**
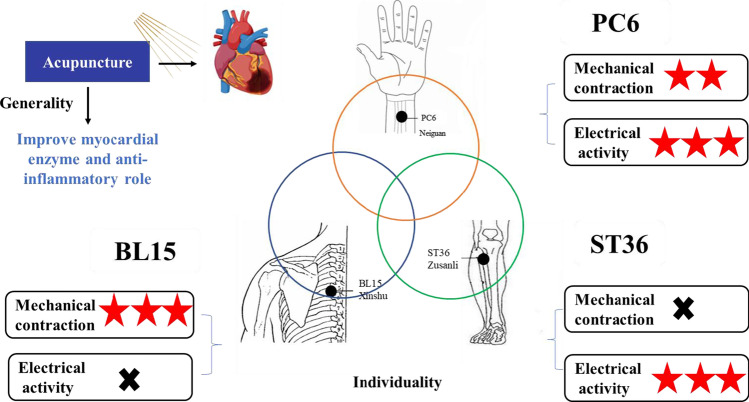
Graphical abstract of the mechanism for the protective effect of acupuncture on different acupoints

Among PC6, BL15, and ST36, PC6 significantly promoted systolic function with less incidence of arrhythmia, while BL15 improved systolic function with increased susceptibility to arrhythmia, and ST36 could not improve systolic function. Our study confirmed that acupoint specificity versus acupuncture outcomes had internal correlation with acupoints and meridian specificity. Myocardial ischemia had closer relationship with PC6 (pericardial meridian acupoint-directly heart divergent meridian), than that of BL15 and ST36 (Fig. 6). Our results also provide scientific evidence for the acupoint selection of heart diseases in clinical application. Further research is needed to elucidate the detail mechanism of point-specific actions in acupuncture.

## Supplementary Information

Below is the link to the electronic supplementary material.

Supplementary file1 (DOCX 416 KB)

## Data Availability

The datasets used and/or analyzed during the current study are available from the corresponding author on reasonable request.

## Abbreviations

MI: Myocardial ischemia
LAD: Left anterior descending
ECG: Electrocardiogram
EF: Ejection fraction
FS: Fractional shortening
SV: Stroke volume
HR: Heart rate
CK: Creatine kinase
CK-MB: Creatine kinase-MB
LDH: Lactate dehydrogenase
cTnT: Cardiac troponin T
CVF: Collagen volume fraction
TNF-α: Tumor necrosis factor-α
IL-6: Interleukin-6
IL-10: Interleukin-10
INF-γ: Interferon-γ; P2X7
Serca: Sarco endoplasmic reticulum Ca2 + -ATPase
Cav1.2: Calcium channel, voltage-dependent, L-type, alpha 1C subunit
Col1α: Collagen fiber 1α
Cthrc1: Collagen triple helix repeat-containing protein 1
DEGs: Differentially expressed genes
GO: Gene ontology
KEGG: Kyoto Encyclopedia of Genes and Genomes
IL-5r: Interleukin-5 receptor
Wfdc12: WAP four-disulfide core domain protein 12
Wnt 4: Wingless/Int-1 type protein 1
Doc2b: Double C2 domain beta
Erdr1: Erythroid differentiation regulator1

## References

[1] Roth GA, Mensah GA, Johnson CO, Addolorato G, Ammirati E, Baddour LM (2020). Global burden of cardiovascular diseases and risk factors, 1990–2019: update from the GBD 2019 study. J Am Coll Cardiol.

[2] Anzai T (2018). Inflammatory mechanisms of cardiovascular remodeling. Circ J.

[3] Zhao L, Li DH, Zheng H, Chang XR, Cui J, Wang R (2019). Acupuncture as adjunctive therapy for chronic stable angina: a randomized clinical trial. JAMA Intern Med.

[4] Fu SP, He SY, Xu B, Hu CJ, Lu SF, Shen WX (2014). Acupuncture promotes angiogenesis after myocardial ischemia through H3K9 acetylation regulation at VEGF gene. PLoS One.

[5] Zeng Q, He H, Wang XB, Zhou YQ, Lin HX, Tan ZP (2018). Electroacupuncture preconditioning improves myocardial infarction injury via enhancing AMPK-dependent autophagy in rats. Biomed Res Int.

[6] Hong H, Cao X, Deng T, Meng XM, Li XM, Zhu LJ (2022). Acupuncture at Neiguan suppresses PVCs occurring post-myocardial infarction by alleviating inflammation and fibrosis. Chinese Medicine.

[7] Liu SB, Wang ZF, Su YS, Qi L, Yang W, Fu MZ (2021). A neuroanatomical basis for electroacupuncture to drive the vagal-adrenal axis. Nature.

[8] Torres-Rosas R, Yehia G, Pena G, Mishra P, Thompson-Bonilla MR, Moreno-Eutimio MA (2014). Dopamine mediates vagal modulation of the immune system by electroacupuncture. Nat Med.

[9] Ye YM, Birnbaum Y, Widen SG, Zhang ZH, Zhu SP, Bajaj M (2020). Acupuncture reduces hypertrophy and cardiac fibrosis, and improves heart function in mice with diabetic cardiomyopathy. Cardiovasc Drugs Ther.

[10] Kim SK, Bae H (2010). Acupuncture and immune modulation. Auton Neurosci.

[11] Xiao N, Li Y, Shao ML, Cui HF, Zhang CY, Kong SP (2020). Jiaji (EX-B2)-based electroacupuncture preconditioning attenuates early ischaemia reperfusion injury in the rat myocardium. Evid-Based Complement Alternat Med.

[12] Langevin HM, Wayne PM (2018). What is the point? The problem with acupuncture research that no one wants to talk about. J Alternat Complement Med.

[13] Xing JJ, Zeng BY, Li J, Zhuang Y, Liang FR (2013). Acupuncture point specificity. Int Rev Neurobiol.

[14] Shi L, Fang JL, Zhao JP, Liu GY, Zhao Q, Zhang JL (2017). Comparison of the therapeutic effects of acupuncture at PC6 and ST36 for chronic myocardial ischemia. Evid-Based Complement Alternat Med.

[15] Yang QQ, Mao HF, Chen X, Zhang YJ, Zhang XL, Liu ZZ (2020). Neiguan (PC6)-based acupuncture pretreatment for myocardial ischemia reperfusion injury: a protocol for preclinical systematic review and meta-analysis. Medicine.

[16] Guo HH, Jing XY, Chen H, Xu HX, Zhu BM (2021). STAT3 but not STAT5 contributes to the protective effect of electroacupuncture against myocardial ischemia/reperfusion injury in mice. Front Med.

[17] Yuan ST, Zhang XZ, Bo YL, Li WZ, Zhang HY, Jiang QL (2014). The effects of electroacupuncture treatment on the postoperative cognitive function in aged rats with acute myocardial ischemia-reperfusion. Brain Res.

[18] Wang CN, Liang X, Yu Y, Li YL, Wen XH, Liu M (2020). Electroacupuncture pretreatment alleviates myocardial injury through regulating mitochondrial function. Eur J Med Res.

[19] Huang Y, Lu SF, Hu CJ, Fu SP, Shen WX, Liu WX (2014). Electro-acupuncture at Neiguan pretreatment alters genome-wide gene expressions and protects rat myocardium against ischemia-reperfusion. Molecules.

[20] Fu SP, Hong H, Lu SF, Hu CJ, Xu HX, Li Q (2017). Genome-wide regulation of electro-acupuncture on the neural Stat5-loss-induced obese mice. PLoS One.

[21] Choi EM, Jiang F, Longhurst JC (2012). Point specificity in acupuncture. Chin Med.

[22] Kaul CL, Ramarao P (2000). Renin release and the sympathetic nervous system. Drugs Today (Barc).

[23] Han XK, Gao Y, Yin X, Zhang ZJ, Lao LX, Chen Q, Xu SF (2021). The mechanism of electroacupuncture for depression on basic research: a systematic review. Chin Med.

[24] Langevin HM, Yandow JA (2002). Relationship of acupuncture points and meridians to connective tissue planes. Anat Rec.

[25] Li NC, Guo Y, Gong YN, Zhang Y, Fan W, Yao KF (2021). The anti-inflammatory actions and mechanisms of acupuncture from acupoint to target organs via neuro-immune regulation. J Inflamm Res.

[26] Zhang RY, Zhu BF, Wang LK, Song Y, Zhao JG, Guo Y (2020). Electroacupuncture alleviates inflammatory pain via adenosine suppression and its mediated substance P expression. Arq Neuropsiquiatr.

[27] Chen T, Xiong Y, Long M, Zheng D, Ke H, Xie J (2019). Electro-acupuncture pretreatment at Zusanli (ST36) acupoint attenuates lipopolysaccharide-induced inflammation in rats by inhibiting Ca^2+^ influx associated with cannabinoid CB2 receptors. Inflammation.

[28] Zhang M, Du QG, Yang FB, Guo Y, Hou YL, Zhu PY (2019). Acupuncture at PC6 prevents cardiac hypertrophy in isoproterenol-treated mice. Acupunct Med.

[29] Moreira BR, Duque AP, Massolar CS, Pimentel RL, Mediano MF, Guimaraes TC (2019). Transcutaneous electrical stimulation of PC5 and PC6 acupoints modulates autonomic balance in heart transplant patients: a pilot study. J Acupunct Meridian Stud.

[30] Wang JY, Yao L, Wu XL, Guo Q, Sun SX, Li J (2021). Protection against doxorubicin-induced cardiotoxicity through modulating iNOS/ARG2 balance by electroacupuncture at PC6. Oxid Med Cell Longev.

[31] Stinnett HO, Bishop VS, Peterson DF (1976). Reduction in baroreflex cardiovascular responses due to venous infusion in the rabbit. Circ Res.

[32] Hsu CC, Weng CS, Liu TS, Tsai YS, Chang YH (2006). Effects of electrical acupuncture on acupoint BL15 evaluated in terms of heart rate variability, pulse rate variability and skin conductance response. Am J Chin Med.

[33] Lee NR, Kim SB, Heo H, Lee YH (2016). Comparison of the effects of manual acupuncture, laser acupuncture, and electromagnetic field stimulation at acupuncture point BL15 on heart rate variability. J Acupunct Meridian Stud.

[34] Driessen HE, van Veen T, Boink G (2017). Emerging molecular therapies targeting myocardial infarction-related arrhythmias. Europace.

[35] Yin J, Chen JD (2010). Gastrointestinal motility disorders and acupuncture. Auton Neurosci.

[36] Liu MC, Zhang SL, Gai Y, Xie MZ, Qi QH (2016). Changes in the interstitial cells of Cajal and immunity in chronic psychological stress rats and therapeutic effects of acupuncture at the Zusanli point (ST36). Evid-Based Complement Alternat Med.

[37] Yang FM, Gong YN, Yu NN, Yao L, Zhao X, Hong SH (2021). ST36 acupuncture alleviates the inflammation of adjuvant-induced arthritic rats by targeting monocyte/macrophage modulation. Evid-Based Complement Alternat Med.

[38] Xie DP, Zhou GB, Chen RL, Qin XL, Du JD, Zhang Y (2020). Effect of electroacupuncture at Zusanli (ST36) on sepsis induced by cecal ligation puncture and its relevance to spleen. Evid-Based Complement Alternat Med.

[39] Oh JE, Kim SN (2021). Anti-inflammatory effects of acupuncture at ST36 point: a literature review in animal studies. Front Immunol.

