# Supplementary material for: Comparative Study of Different Acupoints for Treating Acute Myocardial Ischemia in Mice
Source: J Cardiovasc Transl Res. 2023 Jan 23;16(3):644–61. doi: 10.1007/s12265-022-10346-6 (PMC10300188; doi:10.1007/s12265-022-10346-6)

**s-Table.1**. **Evaluation of arrhythmia incidence and animal survival rate.**

| **Group** | **Number** | **Death** | **Survival rate (%)** | **Arrhythmia** | **Arrhythmia rate (%)** |
| --- | --- | --- | --- | --- | --- |
| **Sham** | **10** | **0** | **100%** | **0** | **0%** |
| **MI** | **7** | **0** | **100%** | **2** | **28.60%** |
| **MI+PC6** | **9** | **1** | **88.90%** | **1** | **11.10%** |
| **MI+BL15** | **10** | **2** | **80%** | **4** | **50%** |
| **MI+ST36** | **8** | **0** | **100%** | **2** | **25%** |

**s-Fig. 1** **Time line of the experiment and acupoints location (Neiguan, Xinshu, Zusanli).**


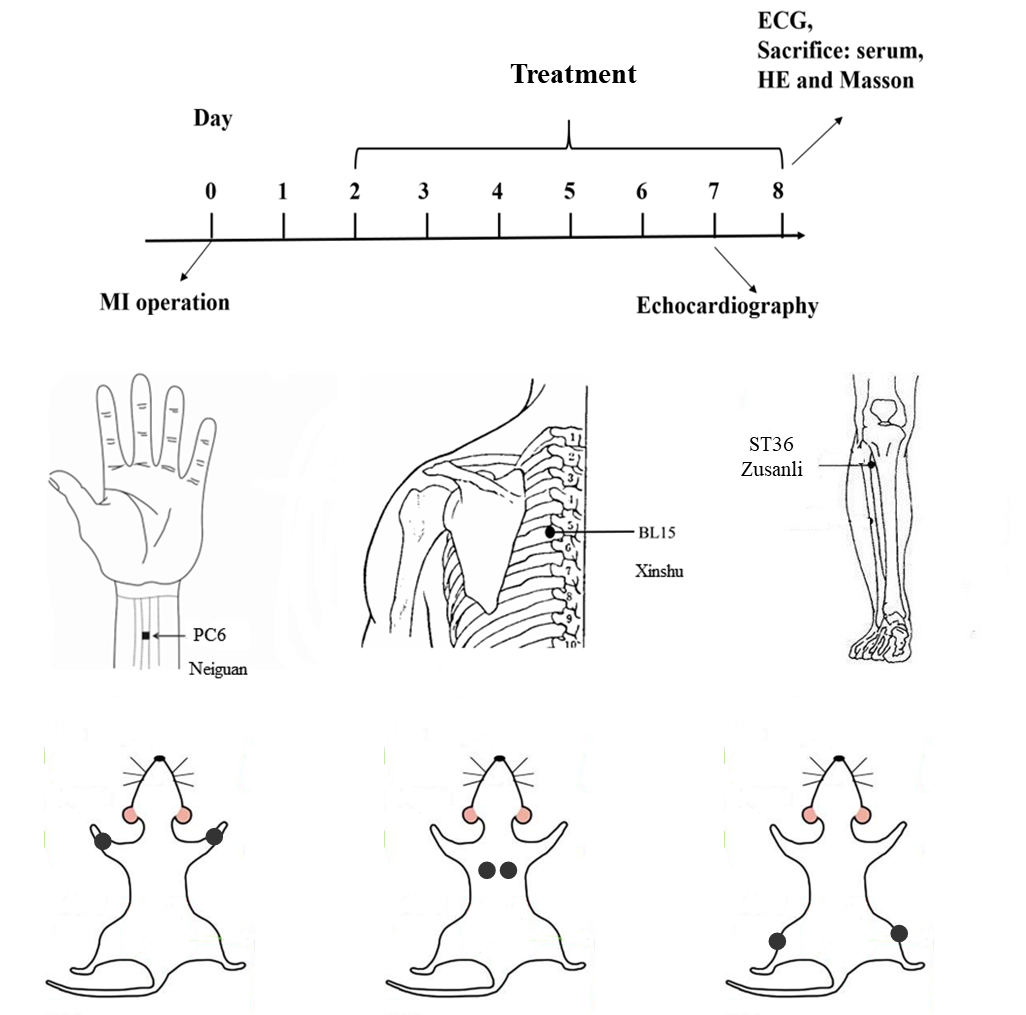


**s-Fig.2** **Serum renin level in each group. B represents *p*<0.05 vs. the MI group.**


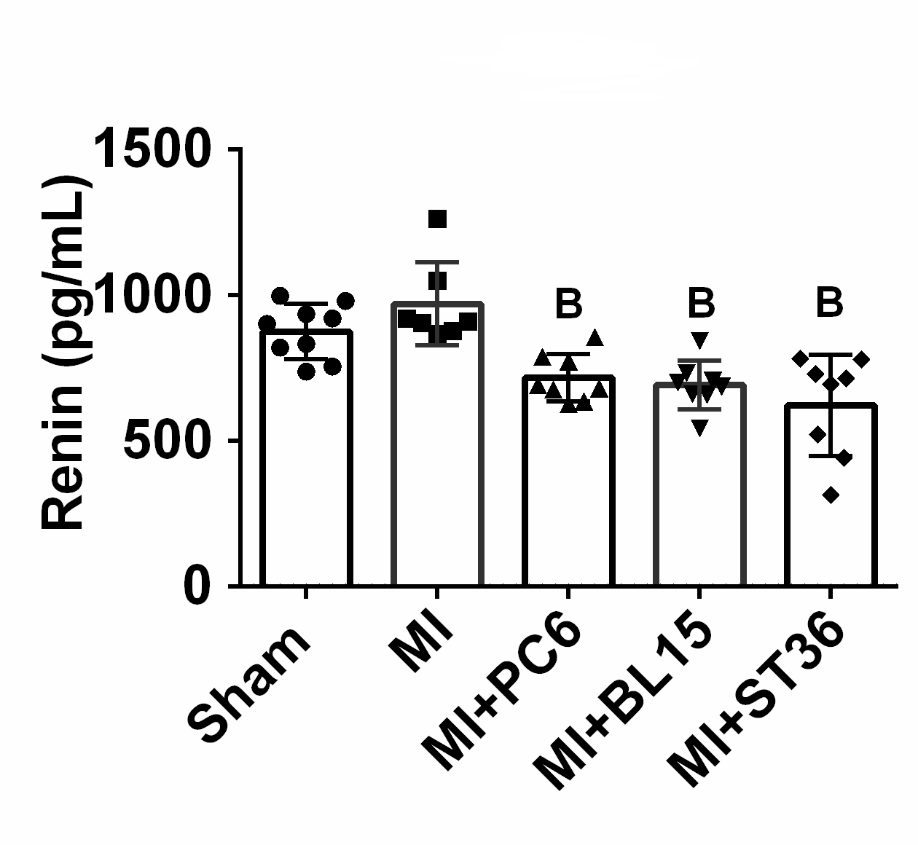

Supplement: Supplementary file 1 — Supplementary file1 (DOCX 416 KB) [file 12265_2022_10346_MOESM1_ESM.docx]
